# Supplementary material for: WordCommentsAnalyzer: A windows software tool for qualitative research
Source: F1000Res. 2018 Sep 4;7:536. Originally published 2018 May 3. [Version 2] doi: 10.12688/f1000research.14819.2 (PMC6137411; doi:10.12688/f1000research.14819.2)
Supplement: Supplementary file 3 [file f1000research-7-17614-s0002.tgz › b2a79496-4ada-47c4-9682-a99ce867836d.zip!/Eight Iranian Health Journals/The Journal of Toloo-e-Behdasht_0.docx]

© The Journal of Toloo-e-Behdasht. This work is licensed under CC BY 4.0 (https://creativecommons.org/licenses/by/4.0/).

Volume & Issue: دوره 17 - شماره 2

Title: بررسی روند تولدها در ایران برحسب گروههای سنی مادران طی سالهای 91-1385

http://tbj.ssu.ac.ir/article-1-1277-fa.html

مقدمه: سن  بارداری از جمله موضوعاتی است که در حال حاضر محققان زیادی به آن پرداخته اند، زیرا با بالا رفتن سن بارداری مشکلات زیادی بوجود می آید که ازمهم ترین آنها می توان به بالا رفتن احتمال تولد نوزاد نارس ویا دارای یک نقص مادرزادی اشاره کرد. هدف از این مطالعه بررسی روند تولد برحسب گروه های سنی مادران طی سالهای 91-1385 بوده است. روش بررسی : در این مطالعه توصیفی-مقطعی داده های مربوط به تعداد تولدها ازمرکز ثبت احوال و داده های جمعیت ازمرکز آمارایران از سال 91-1385 گرفته شد.سپس میزان باروری ویژه سن در           گروه های مختلف سنی مادران محاسبه و از طریق آمار توصیفی و رگرسیون توسط نرم افزار SPSS  تجزیه وتحلیل گردید. یافته ها: نتایج این پژوهش نشان داد که در رده سنی 25 سال مادران به بالا با افزایش زمان شاهد افزایش قابل توجهی در تعداد تولدها هستیم و برعکس در رده های سنی کمتر از 25 سال تعداد تولدها دارای نوسانات رو به کاهش می باشد. نتایج هم چنین نشان داد که میزان باروری ویژه سن نه تنها در سنین بالای مادران بلکه درتمام گروه های سنی افزایش یافته است. نتیجه گیری: با توجه به افزایش میزان باروری ویژه سن و کاهش نسبت تولدها در رده سنی زیر 25 سال می توان نتیجه گرفت که تعداد زنان در رده سنی پایین کاهش یافته است هم چنین افزایش میزان باروری ویژه در تمام رده های سنی نشان می دهد تمایل زنان به داشتن فرزند بیشتر افزایش یافته  است.

Title: بررسی اثر مکمل ال-آرژنین بر شاخص های تن سنجی و ترکیب بدن افراد مبتلا به سندرم متابولیک: یک کارآزمایی بالینی تصادفی کنترل شده

http://tbj.ssu.ac.ir/article-1-2526-fa.html

مقدمه: رشد چشمگیرآمار مبتلایان به سندرم متابولیک در سراسر دنیا و خطر عوارض آن لزوم توجه به یافتن راه های جدیدکنترل آن را مطرح می نماید. مطالعه حاضر به منظور بررسی اثرمکمل یاری ال-آرژنین  برشاخص های تن سنجی  در افراد مبتلا به سندرم متابولیک انجام گرفت. روش بررسی: دراین کارآزمایی بالینی 60  بیمار سندرم متابولیک به طورتصادفی به دوگروه مداخله و کنترل  تقسیم شدند. افراد در گروه مداخله روزانه 5 گرم مکمل-آرژنین  دریافت کردند . وزن، شاخص توده بدن، دور کمر، نسبت دورکمر به باسن، توده چربی و توده بدون چربی بدن  در ابتدا و در پایان 3 ماه  مداخله ارزیابی گردید. یافته ها :در پایان مداخله متغیرهای مربوط به ترکیب بدن تفاوت معنی داری را در بین گروه دریافت کننده مکمل ال-آرژنین و دارونما نشان ندادند(05/0<P). در پایان مداخله  نسبت به ابتدای مطالعه کاهش معنی داری در میانگین درصد چربی بدن(12/9±80/37 به 74/8±26/37 :01/0P=) و افزایش معنی دار در توده بدون چربی بدن (47/4±76/27 به 92±35/28 :01/0 P=)در گروه مکمل ال-آرژنین مشاهده شد اما پس از محاسبه میانگین و انحراف معیار تغییرات متغیرها نسبت به ابتدای مطالعه در بین دو گروه و مقایسه   ان ها نشان داد که تفاوت معنی داری در میزان تغییرات متغیر ها در دو گروه دریافت کننده مکمل ال-ارژنین و دارونما وجود نداشته است(05/0<P). نتیجه گیری:مطالعه ما نشان می دهد که 12 هفته مصرف مکمل ال- آرژنین به میزان 5 گرم در روز  اثر معنی داری در وزن ، شاخص توده بدنی، و ترکیب بدن بیماران سندرم متابولیک نداشت.

Title: بررسی سازه‌های تئوری رفتار برنامه ریزی شده در پیش بینی قصد و رفتار کاهش وزن در نوجوانان مبتلا به اضافه وزن و چاقی

http://tbj.ssu.ac.ir/article-1-2421-fa.html

مقدمه: شیوع اضافه وزن و چاقی عملاً در تمام جوامع و گروه های سنی در جهان با سرعتی هشدار دهنده در حال افزایش است. هدف از مطالعه حاضر بررسی سازه های تئوری رفتار برنامه ریزی شده در پیش بینی قصد و رفتار کاهش وزن در نوجوانان مبتلا به اضافه وزن و چاقی می باشد. روش بررسی: در یک مطالعه توصیفی- تحلیلی به صورت مقطعی در سال 1395،  با استفاده از روش نمونه گیری تصادفی ساده  125 نفر از  نوجوانان 13 تا 18 سال مبتلا به اضافه وزن و چاقی، مراجعه کننده به درمانگاه اطفال واقع در پژوهشکده قلب و عروق اصفهان مورد بررسی قرار گرفتند. ابزار جمع آوری داده ها، پرسشنامه محقق ساخته، منطبق با سازه های تئوری رفتار برنامه ریزی شده بود، که روایی و پایایی آن مورد تأیید قرار گرفت. آنالیز آماری داده ها با استفاده از  نرم افزار  SPSS  نسخه 20 و آزمون های آماری ضریب همبستگی پیرسون و رگرسیون خطی،  مورد تجزیه و تحلیل قرار گرفت. یافته­ ها: میانگین سنی نمونه ها  53/1 ± 20/15سال  بود. نتایج مطالعه نشان می دهد یک همبستگی مثبت و معنی دار  بین کلیه سازه های تئوری رفتار برنامه ریزی شده، با قصد و رفتار کاهش وزن در نوجوانان وجود دارد. سازه های تئوری رفتار برنامه ریزی شده، 38 % قصد و 44 %  رفتار را پیشگویی می کردند. کنترل رفتار درک شده و سپس  هنجار های انتزاعی و نگرش ، مهم ترین متغیر های پیش گویی کننده قصد بودند. نتیجه گیری: یافته های این مطالعه نشان می دهد که ارتقاء کنترل رفتاری درک شده نوجوانان مبتلا به اضافه وزن و چاقی، برای کاهش وزن باید به عنوان اولویت های برنامه های ارتقاء دهنده سلامت در نظر گرفته شود. هم چنین تئوری رفتار برنامه ریزی شده می تواند به عنوان یک چهار چوب مناسب جهت برنامه ریزی  مداخلات آموزشی کاهش وزن ، در نوجوانان مبتلا به اضافه وزن و چاقی به کار رود.

Title: بررسی تاثیر کیفیت خدمات بیمارستان بر رفتار رویگردانی بیماران بستری در بیمارستان شهید صدوقی یزد

http://tbj.ssu.ac.ir/article-1-2755-fa.html

مقدمه: اگر بیماران از بیمارستان رضایت نداشته باشند، این امر منجر به رویگردانی بیمار و ترک رابطه بیمار با بیمارستان­ می­شود. در این شرایط بیماران، بیمارستانی­ را انتخاب می­کنند که خدماتی با بهترین کیفیت را عرضه کند. هدف از این مقاله، بررسی تاثیر کیفیت خدمات بیمارستان بر رفتار رویگردانی بیماران می­باشد. روش بررسی: پژوهش، از نوع توصیفی بوده و به صورت مقطعی و کاربردی در بیمارستان شهید صدوقی یزد که بزرگ­ترین بیمارستان در سطح استان یزد می­باشد انجام شد. جامعه آماری کلیه بیماران بستری شده در شش­ماهه اول سال 1395 بود. روش نمونه­گیری، تصادفی ساده بود و حجم نمونه توسط فرمول کوکران، 196 تعیین شد. داده­ها با استفاده از دو پرسش­نامه بسته، جمع­آوری و تحلیل داده­ها با استفاده از نرم افزار آماریSPSS  نسخه 19 صورت گرفت. در تجزیه و تحلیل داده­ها از شاخص­های مرکزی و پراکندگی و آزمون­های همبستگی و رگرسیون استفاده شد. یافته­ها: میانگین کیفیت خدمات بیمارستان 025/1±508/3 و میانگین رفتار رویگردانی بیماران 997/0±186/2 به دست آمد. نتیجه آزمون همبستگی پیرسون نشان داد همبستگی منفی و معنی­داری (746/0- r = و000/0p=) بین کیفیت خدمات بیمارستان با رفتار رویگردانی وجود دارد. از میان ابعاد شش­گانه کیفیت خدمات بیمارستان، بعد تواضع بیشترین میزان همبستگی را با رفتار رویگردانی بیماران دارد. هم­چنین نتیجه آزمون رگرسیون نشان داد کیفیت خدمات بیمارستان بر رفتار رویگردانی بیماران تاثیر منفی و معنی­دار دارد. نتیجه­ گیری: با توجه به تاثیر منفی و معنی­دار کیفیت خدمات بیمارستان بر رفتار رویگردانی بیماران، می­توان گفت هر چه مدیران بیمارستان در راستای ارتقاء کیفیت خدمات بیمارستان بکوشند، بیماران در صورت نیاز به مراجعه مجدد، دوباره همین بیمارستان را انتخاب می­کنند. با توجه به همبستگی بالای بعد تواضع با رفتار رویگردانی، می­توان دریافت میزان ادب و احترام کارکنان بیمارستان در برخورد با بیماران، از نظر بیماران بسیار اهمیت دارد.

Title: تاثیر درمان فعال سازی رفتاری بر کاهش علائم افسردگی نوجوانان

http://tbj.ssu.ac.ir/article-1-2756-fa.html

مقدمه: قشر نوجوان، قشر اصلی و آسیب‌پذیر جامعه بوده که آینده جوامع را می‌سازند، از همین رو روانشناسان و متخصصان تعلیم و تربیت از دیرباز به بررسی عوامل مؤثر در بهداشت روانی آن‌ها پرداخته‌اند. یکی از عوامل مؤثر و اساسی در سلامت روان نوجوانان افسردگی است. بر همین اساس این پژوهش با هدف بررسی تاثیر فعال سازی رفتاری بر کاهش افسردگی نوجوانان انجام شده است. روش بررسی: این پژوهش به روش آزمایشی با استفاده از گروه کنترل بود. جامعه موردمطالعه این پژوهش شامل کلیه دختران سال اول دبیرستان دوره متوسطه دوم در سال تحصیلی 94-1393 بود که با استفاده از نمونۀ گیری خوشه‌ای چندمرحله‌ای از بین دانش‌آموزان سال اول متوسطه دور دوم 30 نفر (15 نفر گروه آزمایش و 15 نفر گروه گواه) انتخاب‌شده‌اند. در این پژوهش از پرسشنامه بک  (BDI-II) استفاده‌شده است. برای تحلیل داده‌ها از آزمون تحلیل کوواریانس (آنکوا) استفاده‌شده است. یافته‌ها: نتایج پژوهش نشان داد، 15 جلسه درمان فعال‌سازی رفتاری متناسب با نوجوانان توانسته در علائم افسردگی نوجوانان بین گروه آزمایشی و گواه پس از تعدیل اثر متغیر همپراش (پیش‌آزمون) در پس‌آزمون تفاوت معناداری (30/0=2௰، 001/0 ˂P، 52/61=(1،27)F) ایجاد کند. نمرات میانگین تعدیل‌شده نشان می‌دهد که  گروه آزمایشی بهبود معناداری را در نشانه‌های افسردگی داشته‌اند. نتیجه‌گیری: بر اساس یافته‌های پژوهش، درمان مبتنی بر فعال‌سازی رفتاری به‌عنوان یک روش کاربردی می‌تواند به کاهش نشانه های افسردگی نوجوانان کمک کند و به عنوان یک روش موثر در جلسات درمانی مورد استفاده درمانگران قرار گیرد. با استفاده از این روش می توان نوجوانان افسرده را به عضوی فعال در جامعه تبدیل کرد و مشارکت آنها را در فعالیت های اجتماعی بهبود بخشید

Title: ارزیابی ارگونومیک ریسک ابتلا به اختلالات اسکلتی عضلانی در نیروهای خدماتی

http://tbj.ssu.ac.ir/article-1-2424-fa.html

مقدمه: اختلالات اسکلتی عضلانی شایع ترین مشکل سلامتی شغلی هستند که منجر به افزایش هزینه‌ها و کاهش بازده‌ی نیروی کار می‌شوند. این مطالعه با هدف ارزیابی شیوع اختلالات اسکلتی عضلانی و ارزیابی سطح ریسک ابتلا به این اختلالات در کارکنان خدماتی انجام شد. روش بررسی: در این مطالعه که بصورت مقطعی تحلیلی انجام شد، 76 نفر از نیروهای خدماتی دانشکده‌ها و خوابگاه‌های دانشجویی دانشگاه علوم پزشکی شهید صدوقی یزد به روش سرشماری انتخاب و مورد بررسی قرار گرفتند. ابزار جمع‌آوری اطلاعات پرسشنامه نوردیک و روش REBA بود. برای توصیف داده‌ها از آمار توصیفی(میانگین، انحراف معیار و جدول توزیع فراوانی)، برای مقایسه میانگین‌ها ازآزمونt  مستقل و جهت مقایسه‌ی شیوع‌ اختلالات اسکلتی عضلانی از آزمون کای اسکوئر استفاده شد. یافته‌ها: میانگین سن و سابقه کار افراد مورد مطالعه به ترتیب برابر با 69/9±28/37 و 17/7±94/11 سال بود.در بین اختلالات مورد بررسی بیشترین شیوع به ترتیب مربوط به ناراحتی در ناحیه کمر (3/51 درصد)، مچ‌پا (7/48 درصد) و زانو‌ها (4/47 درصد) بود. بین میانگین سن و سابقه کار افرادی که دچار اختلال اسکلتی عضلانی بودند در مقایسه با افرادی که دچار اختلال اسکلتی عضلانی نبودند، اختلاف معنادار آماری وجود نداشت. ارزیابی پوسچر REBA نشان داد که بسیاری از شاغلین حین کار وضعیت بدنی نامناسب داشتند. نتیجه‌گیری: یافته‌های این پژوهش حاکی از شیوع بالای اختلالات اسکلتی عضلانی در بین افراد مورد مطالعه می‌باشد و لزوم انجام اقدامات اصلاحی در جهت بهبود شرایط کاری این افراد را یادآوری می‌کند.

Title: بررسی تأثیر آموزش ویژگی‌های زیستی، شناختی و عاطفی دوران بلوغ به مادران بر بهبود روابط والد-فرزندی دختران دارای بلوغ زودرس

http://tbj.ssu.ac.ir/article-1-2047-fa.html

مقدمه: این پژوهش با هدفِ بررسی تأثیر آموزش ویژگی­های زیستی، شناختی و عاطفی دوران بلوغ به مادران بر بهبود روابط والد - فرزندی دختران دارای بلوغ زودرس به اجرا در آمد. روش بررسی: روش پژوهش نیمه آزمایشی و از نوع پیش‌آزمون- پس‌آزمون با گروه کنترل بود. جامعه آماری شامل دانش آموزان دختر مقطع ابتدایی دارای بلوغ زودرس منطقه فلاورجان اصفهان بودند که با روش نمونه­گیری تصادفی خوشه­ای از بین کلیه مدارس ابتدایی دخترانه منطقه فلاورجان که شامل 35 مدرسه بود؛ 6 مدرسه به صورت تصادفی انتخاب شد و از بین کلیه دانش­آموزان دختر دارای بلوغ زودرس جنسی که دارای سن 11 سال و کمتر بودند، 30 نفر در دو گروه آزمایش و کنترل گمارش شدند. بعد از آن مادران دانش­آموزان گروه مداخله در طول 8 جلسه آموزش ویژگی­های زیستی، شناختی و عاطفی دریافت کردند. اﺑﺰار­ﮔﺮدآوری اﻃﻼﻋﺎت، ﭘﺮﺳﺸﻨﺎﻣﻪ رابطه والد - فرزندی ﺑﻮده ﻛﻪ ﺑﻪ ﻣﻨﻈﻮر ارزﻳﺎﺑﻲ ﺗأﺛﻴﺮ ﻣﺪاﺧﻠﻪ، در دو ﻣﺮﺣﻠﻪ ﻗﺒﻞ و  ﺑﻌﺪ از ﻣﺪاﺧﻠﻪ ﺗﻮﺳﻂ داﻧﺶ آﻣﻮزان ﺗﻜﻤﻴﻞ ﮔﺮدﻳﺪ. یافته­ها: نتایج نشان داد آموزش ویژگی‌های زیستی، شناختی و عاطفی دوران بلوغ به مادران اثر معناداری بر کیفیت رابطه والد- فرزندی داشت. هم چنین نتایج نشان داد آموزش ویژگی‌های زیستی، شناختی و عاطفی دوران بلوغ به مادر به ‌طور اختصاصی­تر بر احساسات مثبت، همانندسازی با مادر و میزان ارتباط در گروه آزمایشی تأثیرمعنی‌دار دارد(01/0>p) و بر مؤلفه عدم تنفر و گم‌گشتگی نقش در رابطه والد -فرزندی تأثیر معنی‌داری ندارد. نتیجه­گیری: آموزش ویژگی‌های زیستی، شناختی و عاطفی دوران بلوغ به مادران می­تواند روابط والد – فرزندی را در دخترانِ بلوغ زودرس بهبود بخشد.

Title: شناسایی ارزشهای کاربران ترافیک در شهر یزد و ارتباط آن بر رفتار ترافیکی آنها (با تمرکز برکاهش تلفات وحوادث رانندگی)

http://tbj.ssu.ac.ir/article-1-2759-fa.html

مقدمه : تصادف وسایل نقلیه و موتوری یکی از عمده‌ترین دلایل مرگ و میر در سطح جهانی هستند و بالاترین نرخ این مرگ و میر به کشورهای در حال توسعه تعلق می‌گیرد. به طور متوسط هر روزه سه هزار تصادف خودرویی منجر به مرگ در سراسر جهان اتفاق می‌افتد و مرگ‌های مرتبط با وسایل نقلیه یازدهمین عامل رایج مرگ‌ و ‌میر در کشورهای در حال توسعه هستند. این مطالعه با هدف شناسایی ارزشهای کاربران ترافیک در شهر یزد و ارتباط آن بر رفتار ترافیکی آنها انجام گرفت. روش بررسی: این پژوهش از نظر روش توصیفی-تحلیلی ، از نظر هدف کاربردی ، مقطع زمانی آن ابتدای سال 96 و جامعه آماری آن کلیه  رانندگان شهر یزد بودند که بر حسب جدول کرجسی و مورگان  384 نفر به عنوان نمونه به صورت تصادفی انتخاب شدند.  بدین صورت که شهر یزد به 5 بخش تقسیم شده و پرسش گر در هر بخش به صورت تصادفی 77 نفر را از بین رانندگان مختلف از لحاظ سن و نوع وسیله در آن بخش انتخاب نموده و پرسشنامه را بعد از دادن توضیحات لازم جهت پر کردن در اختیار آنها قرار داده است.  سوالات پرسشنامه بر اساس ارزش های کاربران ترافیک در یزد بود  براساس مطالعه مبنایی، ادبیات و تلفیق آن با نتایج به دست آمده از اطلاعات تحقیقات میدانی پژوهش حاصل شده بود. برای طبقه بندی و تجزیه و تحلیل داده ها از نرم افزارهای EXCEL و 23SPSS   استفاده شد که با دادن دو جدول خلاصه مدل (Model Summary) و جدول ضرایب رگرسیونی (ANOVA)، مقادیر همبستگی بین دو متغیر(Rو R2) و سطح معنا داری(sig) مشخص شده و نتایج به دست آمده از آنها مورد تحلیل قرار گرفت. یافته ها: متغیرهای ارزش مذهبی (289/0)، ارزش اقتصادی (282/0)، ارزش اجتماعی (250/0)، ارزش دانش (199/0)، ارزش سلامتی (159/0) و ارزش وجهه خانوادگی (146/0) به ترتیب بالاترین تاثیر را روی متغیر رفتار ترافیکی داشته اند و متغیرهای ارزش مردمی، زیبایی شناختی، لذت جویانه و قدرت، تاثیر معناداری نداشته است. نتیجه گیری: با تکیه بر ارزشهایی که در یک جامعه وجود دارد می توان به نوعی همبستگی در جهت پیش بردن و برجسته کردن ارزشهای حاکم رسید، لذا رعایت ارزشهای مذکور برابر نتایج به دست آمده با توجه به همسویی با رفتارهای ترافیکی مناسب و درست باعث رعایت کردن قوانین و مقررات راهنمایی و رانندگی شده که این امر موجب نظم بخشی به ترافیک و کاهش تصادفات و تلفات  ناشی از آن می شود.
